# Characterizing the immunoprofile and endogenous immune response to squamous cell carcinomas of the head and neck to guide development of effective immunotherapy strategies

**DOI:** 10.1186/2051-1426-2-S3-P247

**Published:** 2014-11-06

**Authors:** Tarsem Moudgil, R Bryan Bell, Rom Leidner, Zipei Feng, Christopher Paustian, Christopher Dubay, Traci L Hilton, Brendan Curti, Walter J Urba, Carlo B Bifulco, Hong-Ming Hu, Bernard A Fox

**Affiliations:** 1 Robert W. Franz Cancer Research Center, Earle A. Chiles Research Institute, Providence Cancer Center, Portland, OR, USA; 2 Oral, Head and Neck Cancer Program and Clinic, Providence Cancer Center, Portland, OR, USA; 3 Molecular Microbiology and Immunology, OHSU, Portland, OR, USA; 4 UbiVac, Portland, OR, USA

## 

Squamous cell carcinoma of the head and neck (HNSCC) is the 6^th ^leading cause of cancer by incidence worldwide with approximately 600,000 new cases per year. Unfortunately, only 40-50% of these patients will survive for 5 years. In order to study the immune response in patients with this disease we have developed a HNSCC tumor bank to compliment our Oral, Head and Neck Cancer Program. This tumor bank is cryopreserving enzymatically isolated viable cells from resected tumors (n = 128). We are also attempting to develop primary cell lines (10 lines established) and are isolating and assessing tumor-specific function of tumor-infiltrating lymphocytes (TIL) (n = 49). Since HNSCC can express immune inhibitory molecules and secrete suppressive factors, we are developing single stains and multi-spectral imaging protocols to assess the immune contexture of the tumor microenvironment by immunohistochemistry and immunoflouresence and are overlaying these studies with T cell functional activity and ultimately, with clinical outcome. Preliminary analyses suggest that tumor-specific T cells can be detected in 68% (N = 16) of patients evaluated. A goal of these studies is to identify strategies that will allow tailoring of therapy for patients with HNSCC. One component is to identify which inhibitors are present in a given tumor. Since not every tumor appears to contain TIL capable of recognizing autologous tumor, strategies to prime tumor-specific T cells represents another area of interest. DPV-001 is a microvesicle vaccine, DRibbles, that contains an average of at least 66 proteins that are overexpressed by HNSCC (TCGA provisional RNASeq n = 303 pts). The vaccine also contains multiple DAMPs and agonist activity for TLR 2, 3, 4, 7 and 9 packed into stable double membrane microvesicles that are targeted to CLEC9A+ APC. To increase potential activity against HPV positive cancers we have developed a mosaic construct encoding E6 and E7 peptides for a number of HPV strains and are evaluating both protein and gene-based HPV vaccine strategies. We are using the HNSCC TIL lines to evaluate DRibble vaccines and potential for adoptive immunotherapy trials.

Support: NCI U43CA165048 and R44 CA121612 (TLH), OMS (HH, RBB), Steve and Cindy Harder, Robert W. and Elsie Franz, Wes and Nancy Lematta, Lynn and Jack Loacker, and The Chiles foundation (BAF).

## Consent

Written informed consent was obtained from the patient for publication of this abstract and any accompanying images. A copy of the written consent is available for review by the Editor of this journal.

